# Molecular docking analysis of phytocompounds from Acacia farnesiana with protein targets linked to bronchitis

**DOI:** 10.6026/97320630017557

**Published:** 2021-05-31

**Authors:** Mallikarjun S Beelagi, Manoj Manjunath Bongale, Anisha S Jain, Kollur Shiva Prasad, Sharanagouda S Patil, Govindappa Mellappa, Chandan Dharmashekar, P Ashwini, R Triveni, Chandan Shivamallu, Chandrashekar Srinivasa

**Affiliations:** 1 Department of Biotechnology and Bioinformatics, School of Life Sciences, JSS Academy of Higher Education and Research, Mysuru, Karnataka 570015, India; 2 Department of studies in Biotechnology, Davangere University, Davangere 577007, Karnataka, India; 3 Department of Sciences, Amrita School of Arts and Sciences, Amrita Vishwa Vidyapeetham, Mysuru Campus, Mysuru, Karnataka 570 026, India; 4 ICAR, National Institute of Veterinary Epidemiology and Disease Informatics (NIVEDI), Yelahanka, Bengaluru 560064, India; 5 Department of Studies in Botany, Davangere University, Davangere 577007, Karnataka, India; 6 Department of Microbiology, School of Life Sciences, JSS Academy of Higher Education and Research, Mysuru, Karnataka–570 015, India; 7 Sri HN Ananth Kumar, P.U. College, Bidadi 562109, Karnataka, India

**Keywords:** Acacia farnesiana, acute bronchitis, molecular docking, discovery studio, phytocompounds

## Abstract

Acute bronchitis is a lower respiratory tract lung infection that causes bronchial inflammation. The known protein drug targets are peptidoglycan D, D-transpeptidase, and DNA topoisomerase 4 subunit A for bronchitis linked infections. These are the membrane associated macromolecules which takes a major role in the formation of cell wall membrane by synthesising the cross-linked peptidoglycan. Therefore, it is of interest to design molecules with improved binding features with these protein targets. Hence, we document the molecular docking analysis data of four phytocompounds from Acacia farnesiana having optimal binding features with these targets linked to bronchitis for further consideration.

## Background

**Figure 1:**
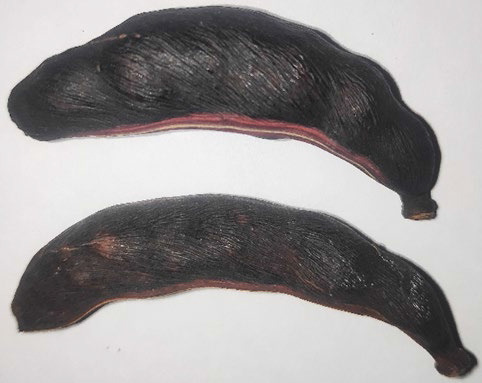
Natural image of Acacia farnesiana fruit.

**Figure 11:**
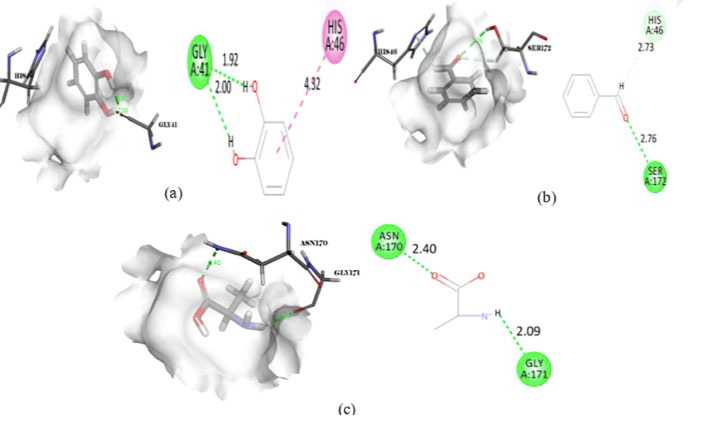
Intermolecular interaction of DNA topoisomerase 4 subunit with (a) Pyrocatechol, (b) Benzaldehyde, (c) DL-Alanine-15N

More than 50% patients of are exposed to hospital borne bronchitis linked infections [1]. Data in the drug bank database shows that the known protein targets for bronchitis [2, 3] are the penicillin-binding proteins (PBP) [4-8], Peptidoglycan-D D-transpeptidase [9, 10], DNA topoisomerase 4 subunit A [11-14] and DNA gyrase subunit [15]. Therefore, it is of interest to document the molecular docking analysis of phytocompounds from Acacia farnesiana (Figure 1-Figure 11) with the known protein targets (Table 1 - see PDF) linked to bronchitis.

## Materials & Methods:

### Target protein, sequences, structures, data preparation and validation:

The (1) Penicillin-binding protein 1A (PDB ID: 2WAF), (2) Peptidoglycan D, D-transpeptidase (PDB ID: 6HZQ), E.coli, (3) Penicillin-binding protein 1B (PDB ID: 3VMA) and (4) Penicillin-binding protein 1A(PDB: 2ZC6), were downloaded from the PDB database. The 3-dimensional structure models for DNA gyrase subunit A, DNA topoisomerase 4 subunit A and peptidoglycan D-D-transpeptidase (Clostridium perfringens (strain 13 / Type A)) developed using the Swiss-Model and Phyre2 web tool with sequences downloaded from the UniPort database. The Ramachandran plots [16] were drawn for the models (Table 2 - see PDF).

### Ligand preparation and validation:

2D and 3D data on phytochemicals from Acacia farnesiana (sweet acacia) were retrieved from IMPPAT (Indian Medicinal Plants, Phytochemistry and Therapeutics) in data formats such as PDB, sdf, mol, and pdbqt. Acacia farnesiana has a total of 23 types of phytochemicals including flavonoids and other derivatives. We used 18 phyto chemicals excluding 5 flavonoids for this study.

### Molecular docking:

Discovery Studio Client Version 20.1 (-CDOCKER Module) is used for molecular docking analysis of protein targets with the selected ligands.

### Known pharmacology properties of DL arginine, decanal, pyrocatechol, sulfoxide, benzaldehyde:

L-arginine is a precursor to nitric oxide or NO and it is synthesized from L-arginine using the enzyme nitric oxide synthase [17-18]. The naturally derived decanal has shown the capability of disrupting the permeability barrier of the cell membrane and it is responsible for the loss of chemiosmotic control [19-21 - check with author]. Pyrocatechol of Aloe vera extraction was exhibited to have maximum antibacterial activity [22]. Sulfoxide in the form of dimethyl sulfoxide (DMSO) acts as an antibacterial and an anti-inflammatory agent against several bacteria such as methicillin-resistant Staphylococcus aureus (MRSA) and Pseudomonas aeruginosa [23]. Benzaldehyde releases intracellular constituents and interacts with the cell surface; induce cell death by causing disintegration of the cell membrane [24,25].

## Results and Discussion:

The bronchitis affects the quality of human community [26]. Penicillin-binding proteins 1B, 1A and Peptidoglycan D-D- transpeptidase, DNA topoisomerase 4 subunit A and DNA gyrase subunit A were taken as protein of target based on their role in disease-causing mechanism. 18 phytochemicals of Acacia farnesiana that have an anti-inflammation are docked and analyzed using Discovery studio v20.1 software with CHARMM module based on the -C-DOCKER energy. Seven phytochemicals of Acacia farnesiana have optimal interaction and binding energy with the seven-targeted proteins (Table 3, Table 4 and Table 5 - see PDF). The high -C-DOCKER energy shows high affinity of the compounds with targets [27 - check with author]. We observed that Pyridoxal phosphate, DL-Arginine, Decanal, and Sulfoxide have high -C-DOCKER energy compared to the rest of the phytochemicals. Therefore, the -C-DOCKER energy, Hydrogen bond, and the position of ligand in the binding pockets of protein molecules are high for these molecules. Thus, these phytochemicals of Acacia farnesiana have potential modulatory functions against the bronchitis targets for further consideration.

## Conclusion:

We document the molecular docking analysis of four phytocompounds (Pyridoxal phosphate, DL-Arginine, Decanal, and Sulfoxide) from Acacia farnesiana having optimal binding features with targets linked to bronchitis for further consideration.

## Figures and Tables

**Figure 2:**
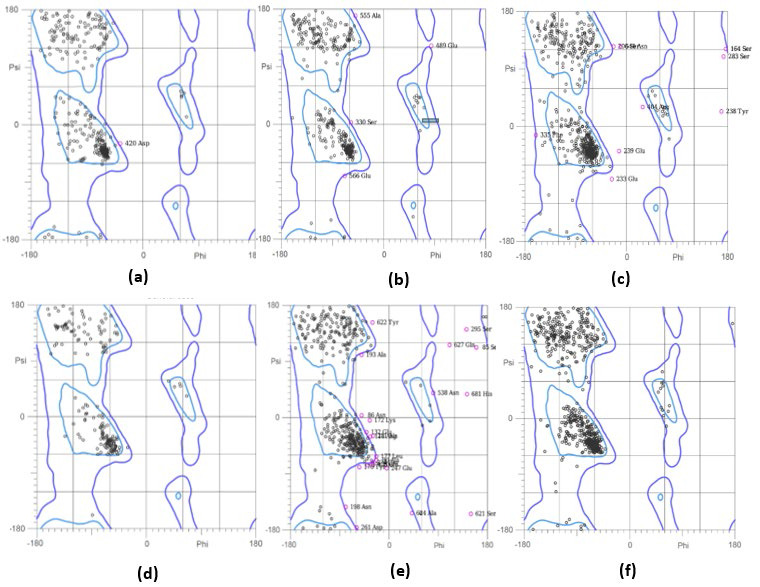
Analysis of Ramachandran plot is shown. (a) DNA topoisomerase 4 subunit, 96.1% (491/511) of all residues were in favored (98%) regions. 99.6% (509/511) of all residues were in allowed (>99.8%) regions. (b) Peptidoglycan_D_D-transpeptidase MrdA 96.4% (535/555) of all residues were in favored (98%) regions. 99.3% (551/555) of all residues were in allowed (>99.8%) regions. (c) Penicillin-binding protein 1B (PDB ID: 3VMA) 92.1% (645/700) of all residues were in favored (98%) regions. 98.3% (688/700) of all residues were in allowed (>99.8%) regions. (d) Peptidoglycan D, D-transpeptidase FtsI (PDB ID: 6HZQ) 95.5% (278/291) of all residues were in favored (98%) regions.99.7% (290/291) of all residues were in allowed (>99.8%) regions. (e) Penicillin-binding protein 1A (PDB ID-2WAF) 83.6% (514/615) of all residues were in favored (98%) regions. 94.8% (583/615) of all residues were in allowed (>99.8%) regions. (f) Penicillin-binding protein 1A (PDB -2ZC6) 95.1% (753/792) of all residues were in favored (98%) regions. 100.0% (792/792) of all residues were in allowed (>99.8%) regions.

**Figure 3:**
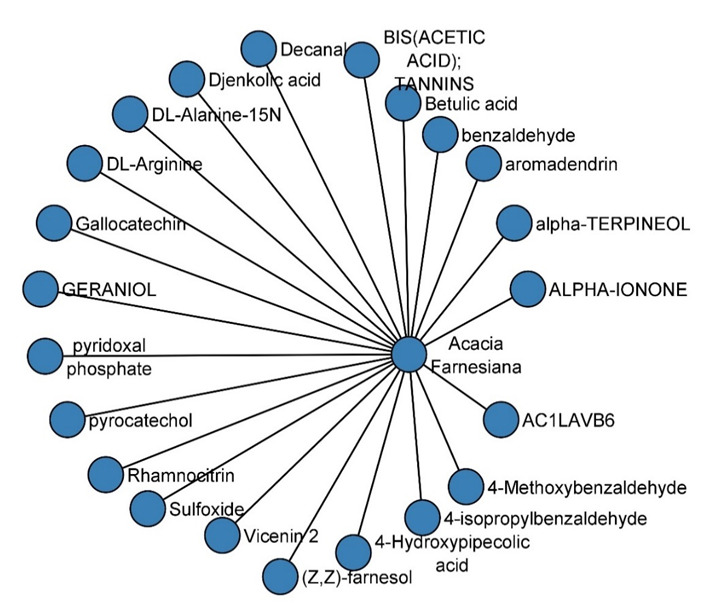
Phytochemicals of Acacia farnesiana retrieved from IMPPAT database.

**Figure 4:**
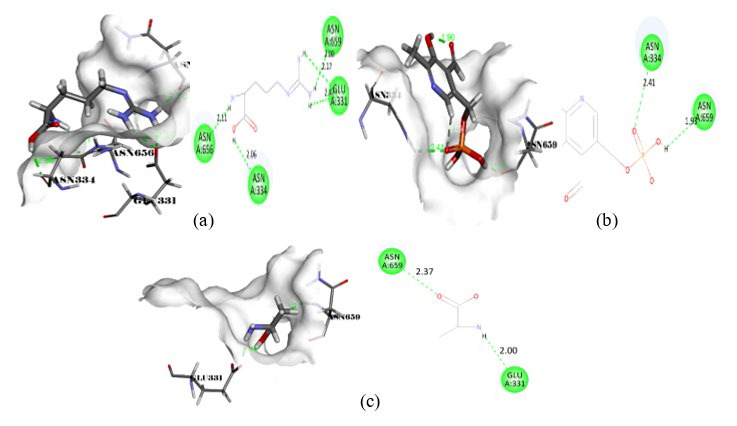
Graphical representation of bioavailability radar of all 18 phytocompounds of Acacia farnesiana.

**Figure 5:**
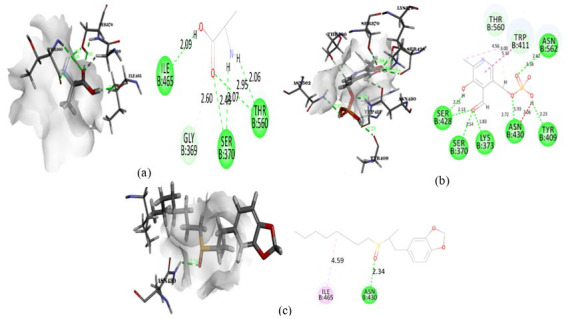
Intermolecular interaction of 2WAF with (a) pyridoxal phosphate, (b) DL-Arginine, (c) Decanal

**Figure 6:**
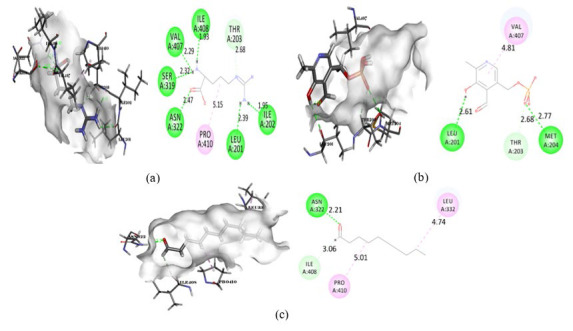
Intermolecular interaction of 2ZC6 with (a) Pyridoxal phosphate, (b) Decanal, (c) Sulfoxide.

**Figure 7:**
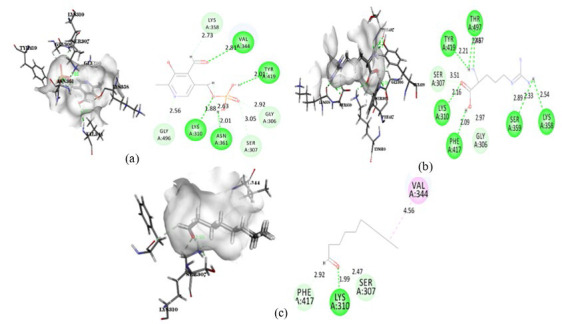
Intermolecular interaction of 3VMA with (a) Pyridoxal phosphate, (b) Decanal, (c) DL-Arginine.

**Figure 8:**
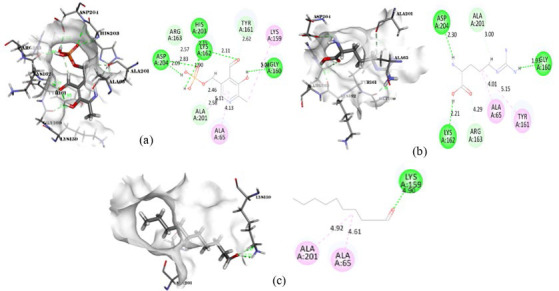
Intermolecular interaction of 6HZQ with (a) Pyridoxal phosphate, (b) DL-Arginine, (c) Decanal

**Figure 9:**
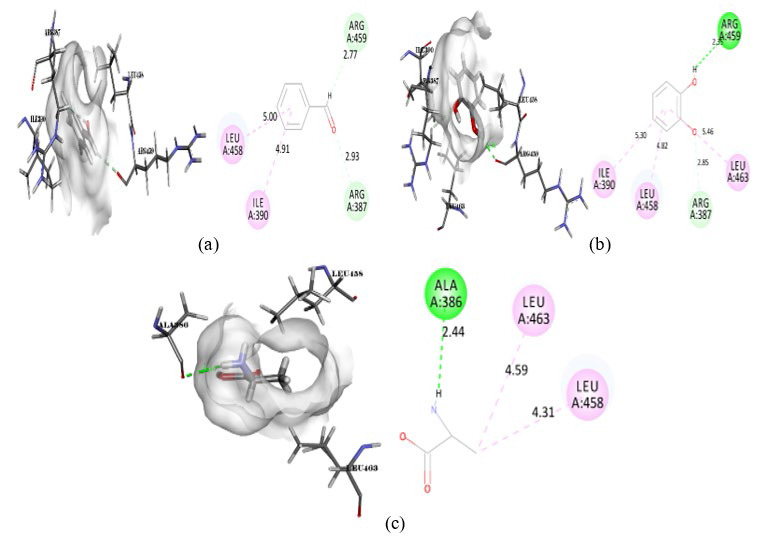
Intermolecular interaction of Peptidoglycan D D-transpeptidase with (a) Pyridoxal phosphate, (b) DL-Arginine, (c) Decanal

**Figure 10:**
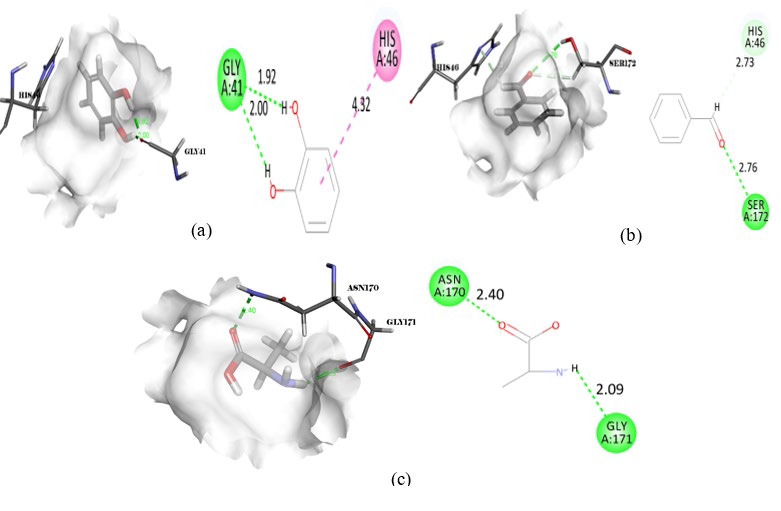
Intermolecular interaction of DNA Gyrase subunit with (a) Benzaldehyde, (b) Pyrocatechol, (c) Alanine-15N

## References

[1] Macfarlane J (2001). Thorax.

[2] https://www.drugbank.ca/.

[3] Prasad SK (2021). Front Chem.

[4] Jain AS (2021). Saudi J Biol Sci.

[5] https://bio.libretexts.org/@go/page/5200.

[6] Macheboeuf P (2006). FEMS Microbiol Rev.

[7] https://bio.libretexts.org/@go/page/5200.

[8] https://www.drugbank.ca/drugs/DB01150.

[9] Lee M (2003). J Am Chem Soc.

[10] https://www.drugbank.ca/drugs/DB01416.

[11] Hiasa H (2018). Methods Mol Biol.

[12] https://www.uniprot.org/uniprot/P43702.

[13] https://www.drugbank.ca/drugs/DB01155.

[14] https://www.drugbank.ca/drugs/DB00978.

[15] Collin F (2011). Appl Microbiol Biotechnol.

[16] http://www.cryst.bbk.ac.uk/PPS95/course/3_geometry/rama.html.

[17] https://go.drugbank.com/drugs/DB00125.

[18] Sepahi M (2017). Iran J Microbiol.

[20] Mahboubi M, Feizabadi M (2009). J ESSENT OIL BEAR PL.

[22] Lawrence R (2009). Braz J Microbiol.

[23] Hassan AS (2014). J Nat Sci Res.

[24] Ullah I (2015). J Microbiol.

[25] Rampogu S (2019). Oxid Med Cell Longev.

[26] Moody L (1990). J Behav Med..

